# Evidence to Suggest That Teeth Act as Human Ornament Displays Signalling Mate Quality

**DOI:** 10.1371/journal.pone.0042178

**Published:** 2012-07-31

**Authors:** Colin A. Hendrie, Gayle Brewer

**Affiliations:** 1 The University of Leeds Institute of Psychological Sciences, Leeds, United Kingdom; 2 School of Psychology, University of Central Lancashire, Preston, Lancashire, United Kingdom; Monash University, Australia

## Abstract

Ornament displays seen in animals convey information about genetic quality, developmental history and current disease state to both prospective sexual partners and potential rivals. In this context, showing of teeth through smiles etc is a characteristic feature of human social interaction. Tooth development is influenced by genetic and environmental factors. Adult teeth record environmental and traumatic events, as well as the effects of disease and ageing. Teeth are therefore a rich source of information about individuals and their histories. This study examined the effects of digital manipulations of tooth colour and spacing. Results showed that deviation away from normal spacing and/or the presence of yellowed colouration had negative effects on ratings of attractiveness and that these effects were markedly stronger in female models. Whitening had no effect beyond that produced by natural colouration. This indicates that these colour induced alterations in ratings of attractiveness are mediated by increased/decreased yellowing rather than whitening per se. Teeth become yellower and darker with age. Therefore it is suggested that whilst the teeth of both sexes act as human ornament displays, the female display is more complex because it additionally signals residual reproductive value.

## Introduction

Ornament displays convey important information about freedom from developmental adversity/disease and other aspects of mate quality to both prospective sexual partners and potential rivals [1], [2], [3], [4], [5], [6]. Individuals displaying high quality characteristics through these displays gain considerable advantage [7]. This is demonstrated within species such as the yellow eyed penguin (*Megadyptes antipodes)* where the higher carotenoid levels seen in healthy animals are reflected in eye and head plumage colouration [8] and associated with increased mating success [9], [10], [11]. Similar effects are seen in a range of other species including house finches *Carpodacus mexicanus* [12], [13] although the relationship between ornamental traits and immune competence is not always a straightforward one [14]. In the context of humans, a variety of ornament displays have been proposed including voice quality [15], waist-to-hip ratio [16] and skin tone [17], [18]. The importance of symmetry [19], [20] and the reproductive advantages associated with height [21] are also well documented.

Several lines of evidence point towards teeth also having function as a human ornament display. Smiling is a common behaviour in our species [22] and this is usually viewed positively by those receiving the smiles [23], [24], [25]. Smiling is seen in many different social situations, including those involving cooperation [26], [27] and affiliation [28]. Importantly, smiling is also one of the first indications of sexual interest in our species [29], [30], [31], [32]. Hence, one of the opening acts of any new sexual partnership is a mutual tooth display.

In further support of this proposal tooth loss is associated with poor general health [33], nutritional deficits [34], cognitive disorder [35], [36], cardiovascular disease [37], stroke [38], [39] and increased risk of death [40]. Absence of teeth may also be indicative of dental caries and periodontitis [41] which are in turn reflective of poor oral hygiene [42], [43] and negative psychological characteristics [44]. Similarly, tooth wear is related to diet, dietary habits [45] and age [46], whilst shape of teeth and spacing may signal the presence of genetic disorders such as Pfeiffer Syndrome [47], [48], Robinow’s Syndrome [49], [50] and Rapp-Hodgkin Syndrome [51].

Tooth colour is also a rich source of information concerning health and genetic quality. Natural colour is mostly determined by the dentine [52]. This is however also influenced by the structural composition/thickness of the enamel and the characteristics of the pellicle layer [53]. Teeth are prone to discolouration from a wide variety of metabolic, inherited and traumatic factors in addition to environmental and dietary causes [54], [55]. In the context of ornament displays it is noteworthy that thinning of the enamel, textural changes and secondary/tertiary deposition of dentine [56] leads to teeth becoming darker and yellower with age [57], [58], [59], [60].

**Figure 1:**
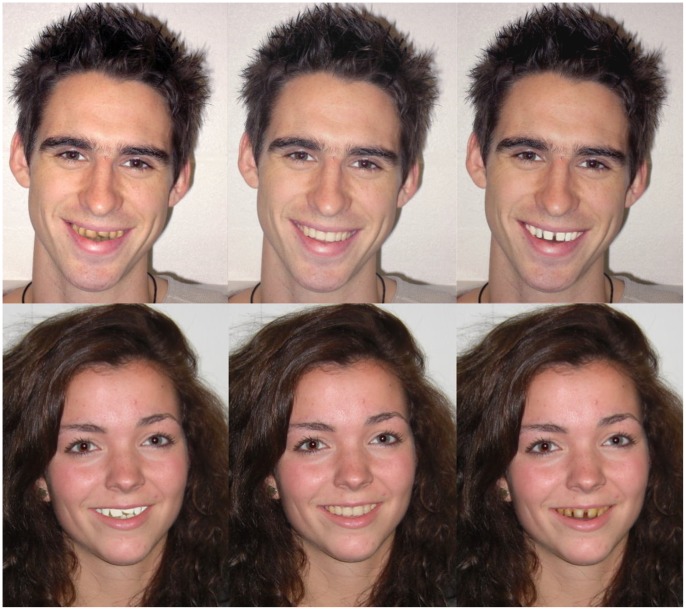
Digital manipulations of tooth spacing and colour. Tooth colour approximated (dark to light) to A4, B1 and OM1 on the Vita Classical shade system whilst naturalistic samples of crowded, normal and widely spaced teeth (shown from left to right) were obtained opportunistically. There were 18 different combinations of tooth colour, spacing and sex of the model. A nested design was used whereby each participant viewed six of these, each shown using a different model (3 of each sex). Stimulus pictures were viewed one at a time and rated independently. See text for further details.

In the US in the order of $1 billion per year is spent on purely cosmetic dental procedures [61] and the desire to improve the appearance of teeth is cross-cultural (e.g. [60], [62], [63], [64]). There are however several components to the tooth display and the relative importance of each of these is not well understood. Therefore the aim of the present studies was to examine the effects of two of these components, spacing and colour. The hypothesis under investigation was that ratings of a model’s attractiveness would be influenced by digital manipulations of their teeth.

## Methods

One hundred and fifty participants (mean = 21.2±1.1 years) were selected by opportunistic sampling (75 males and 75 females). Each participant was presented with pictures of one of six different models (3 males and 3 females) whose own teeth had been digitally replaced with teeth taken from a standard set manipulated by spacing and colour that had been specifically created for the purposes of this study. Picture presentation was made in accordance with a 2 (sex of participant) ×2 (sex of model) ×3 (spacing; crowded, normal and widely spaced) ×3 (colour; yellow, normal and white) **nested** design with colour nested in spacing. There were therefore 9 digitally manipulated photographs of each model that together displayed all combinations of spacing and colour. Each participant thus viewed 6 photographs (3 of each sex) that were selected from each model’s set in randomised counterbalanced order with care being taken to avoid the possibility of order effects and to ensure that no participant viewed the same model twice. All models were Caucasian in origin and aged 20–22 years. Tooth colour was adapted from the Vita Classical shade system [65] approximating to A4, B1, OM1 and photographs of differently spaced teeth were obtained opportunistically. As these were naturalistic images (i.e. photographs), the supernumerary (crowded) teeth were also unavoidably crooked. Examples of these stimulus materials are illustrated in Figure 1.

**Figure 2:**
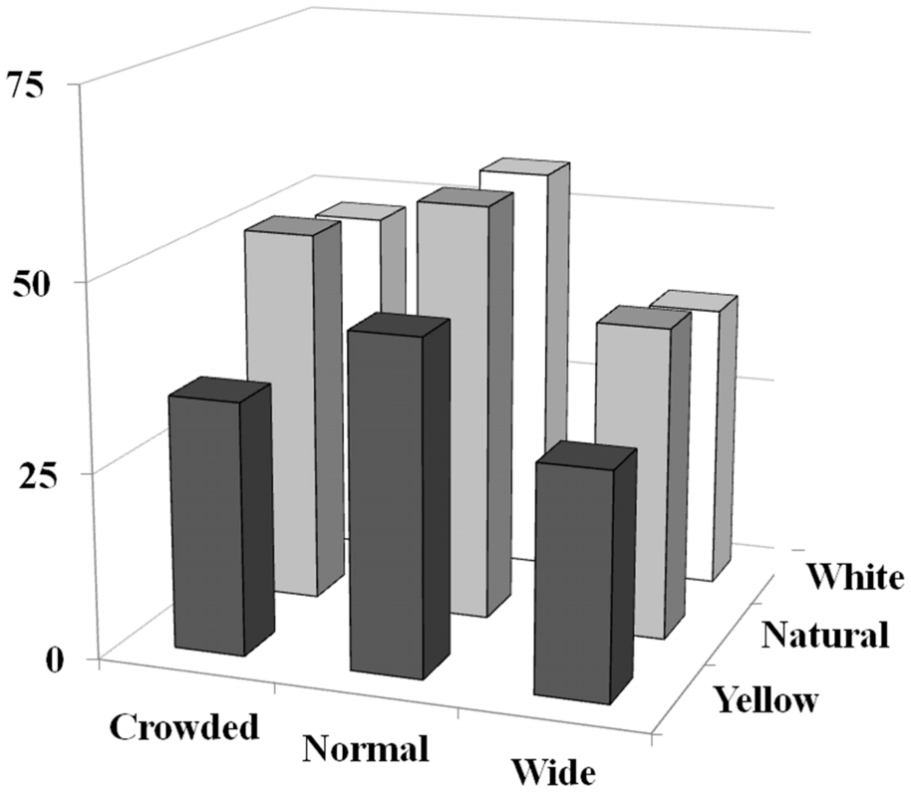
Main effects of digital manipulations of tooth spacing and colour on ratings of attractiveness. Data are expressed as mean ratings of attractiveness given on a 100 mm visual analogue scale. Higher numbers indicate greater attractiveness. Crowded, normal and wide refer to spacing. Yellow, natural and white refer to colour (approximating to A4, B1 and OM1 on the Vita Classical shade scale). Data show that deviations away from normal spacing impact negatively on ratings of attractiveness and that whilst yellowed teeth are rated as least attractive, whitening beyond natural colouration does not further increase ratings of attractiveness. See text for further details.

A set of 10×10 cm cards were produced, printed to a professional standard, laminated and checked that colour integrity matched the Vita Scale described above. Pictures were shown one at a time under ambient indoor workplace lighting and colour remained clearly differentiated. Participants were asked to rate the attractiveness of the person featured in each of the photographs using a 100 mm visual analogue scale. The negative response (i.e. extremely unattractive) was on the left hand side so that higher numbers indicated increased attractiveness when measured from the left. Data were analysed using Statistica (StatSoft**®**).

These studies were approved by The University of Leeds Institute of Psychological Sciences Ethics Committee in accordance with British Psychological Society guidelines. In keeping with these guidelines all participants gave their informed consent in writing prior to beginning the study. Written consent was also obtained from the models used in these studies for their photographs to be digitally manipulated for use in the study and to be used in scientific publications. The models shown in Figure 1 signed a further statement indicating their willingness for the photographs shown in that figure to be published in PLoS ONE.

**Figure 3:**
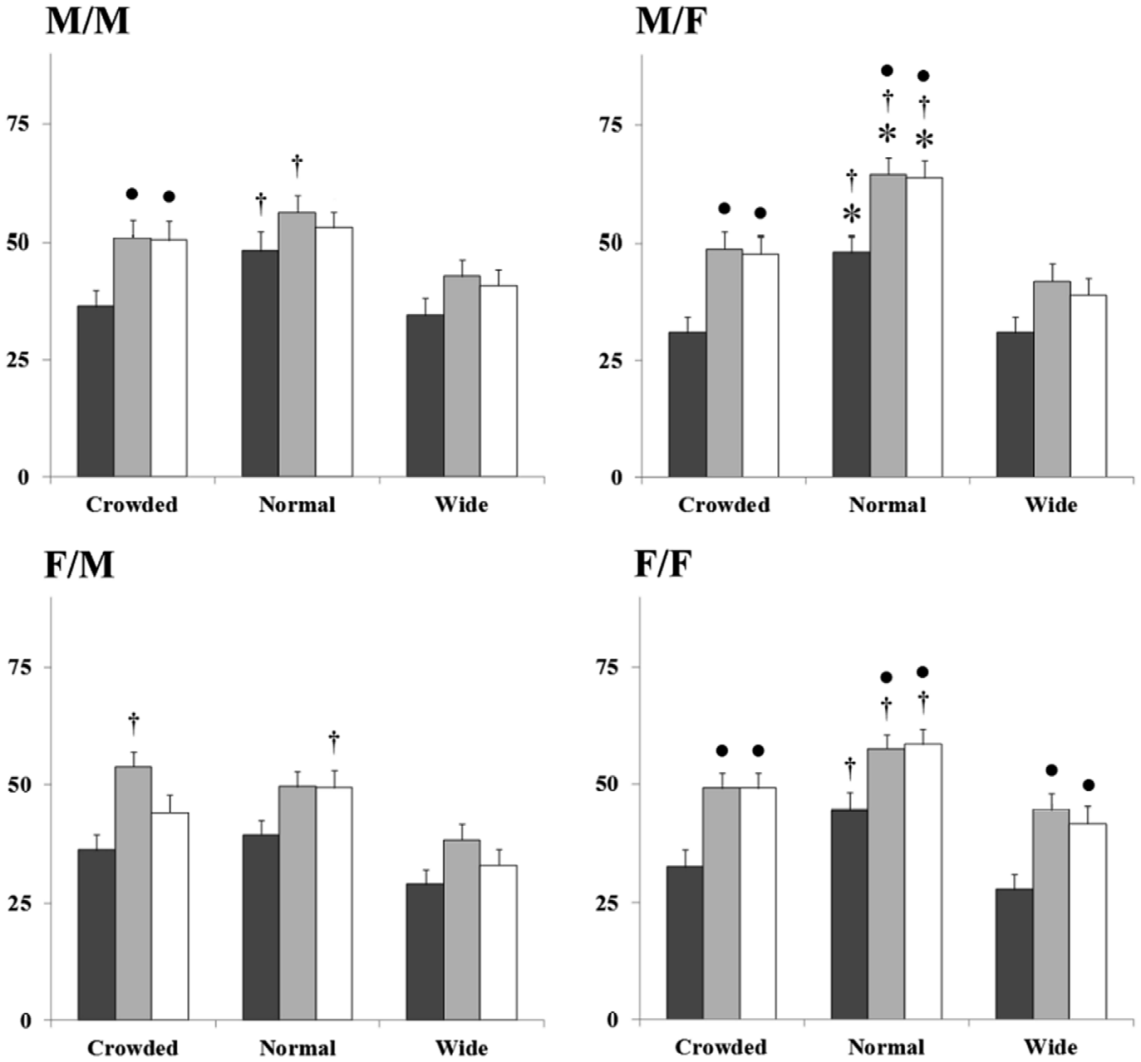
Effects of digital manipulations of teeth on ratings of attractiveness. Data are expressed as mean ratings of attractiveness (± SEM) indicated on a 100 mm visual analogue scale. Higher numbers indicate greater attractiveness. Crowded, normal and wide refer to tooth spacing. Yellow, natural and white colour conditions are indicated by the dark, mid-grey and white bars respectively. **M/M** indicates male participants viewing male models; **M/F**, males viewing females; **F/M**, females viewing males and **F/F**, females rating females. Data indicate that ratings of female attractiveness are more sensitive to manipulations of tooth colour and spacing than males’ regardless of whether male or female participants were doing the rating. The most marked effects in this sex were produced by deviations from normal spacing and yellow colouration. Attractiveness was not increased by further whitening from natural colour (B1 in the Vita shade scale) in any condition * = p<0.01 from crowded teeth in the same colour condition; † = p<0.01 from widely spaced teeth in the same colour condition; • = p<0.01 from yellow teeth in the same spacing condition. See text for further details.

## Results

Analysis of Variance showed significant main effects of teeth spacing (*F*(2, 863) = 62.07, *p*<.01) and colour (*F*(2, 863) = 51.96, *p*<.01). Interactions were not available in consequence of the nested design. Follow up tests were performed using orthogonal contrasts (with α shifted to 0.01 to accommodate the number of contrasts performed) and these revealed that when males viewed other males they rated models with widely spaced teeth as being significantly less attractive than those with normally spaced teeth in the yellow (*F*(1, 863) = 7.15, *p*<.01) and natural (*F*(1, 863) = 7.02, *p*<.01) colour conditions. Males also rated other males with yellow teeth as being significantly less attractive than those with natural (*F*(1, 863) = 8.64, *p*<.01) or white (*F*(1, 863) = 7.77, *p*<.01) teeth in the crowded condition only.

When viewing female models male participants rated those with normally spaced teeth as being significantly more attractive than models with crowded teeth in all colour conditions (yellow (*F*(1, 863) = 13.00, *p*<.01) natural (*F*(1, 863) = 9.55, *p*<.01) white (*F*(1, 863) = 9.21, *p*<.01)). The same effect was seen when comparing normally and widely spaced teeth, again in all colour conditions (yellow (*F*(1, 863) = 12.57, *p*<.01) natural (*F*(1, 863) = 18.21, *p*<.01) white (*F*(1, 863) = 23.42, *p*<.01)). With the focus on colour, yellow teeth were viewed as being significantly less attractive than both naturally coloured and whitened teeth in the crowded (natural (*F*(1, 863) = 14.18, *p*<.01); white (*F*(1, 863) = 11.38, *p*<.01)) and normal (natural (*F*(1, 863) = 10.51, *p*<.01); white (*F*(1, 863) = 9.61, *p*<.01)) spacing conditions.

Few effects were seen when females rated male models, where only those with widely spaced teeth were rated as being less attractive than those with naturally coloured crowded (*F*(1, 863) = 10.73, *p*<.01) and normally spaced whitened teeth (*F*(1, 863) = 10.38, *p*<.01) only.

However, females rating other females viewed models with normally spaced teeth as being significantly more attractive than models with widely spaced teeth in all colour conditions (yellow (*F*(1, 863) = 13.01, *p*<.01) natural (*F*(1, 863) = 8.17, *p*<.01) white (*F*(1, 863) = 11.97, *p*<.01)). This effect was not seen in females with crowded teeth but there was a trend towards this (*p*<0.05 in both the yellow and white colour conditions). With the focus on colour, female participants rated female models with yellow teeth as being significantly less attractive than those with naturally coloured teeth in all spacing conditions (wide (*F*(1, 863) = 14.09, *p*<.01), normal (*F*(1, 863) = 7.69, *p*<.01) crowded (*F*(1, 863) = 11.29, *p*<.01)). Females with yellow teeth were also rated as being less attractive than those with white coloured teeth, again in all spacing conditions (wide (*F*(1, 863) = 8.57, *p*<.01), normal (*F*(1, 863) = 8.45, *p*<.01), crowded (*F*(1, 863) = 11.59, *p*<.01)). These data are summarised in Figures 2 and 3.

## Discussion

Current data show that digital manipulation of tooth colour and spacing produces significant effects on ratings of attractiveness. Deviation away from normal spacing and/or the presence of yellow colouration was found to negatively impact on these ratings. Data further suggest that ratings of attractiveness of female faces are more sensitive to digital manipulation than male faces regardless of which sex is doing the rating.

The signals produced by tooth displays in humans are complex. Smile type, duration and sequence are of major significance for social communication in our species [22], [24], [66], [67]. These signals are also of importance because teeth have several key features that make them ideal vehicles with which to convey to both potential mates and rivals information about genetic quality, developmental history and current disease state (i.e. to serve as ornament displays).

Firstly, genetic factors have a clear influence on the expression and incidence of dental anomalies (e.g. [68], [69], [70]). Secondly, odontogenesis is a multidimensional process that is also sensitive to environmental insults that can then go on to have macroscopic outcomes (for review see [71]). These insults can be reflected in a number of different ways including position along the supernumerary/hypodontia/megadontia/microdontia continuum [72] which is a feature particularly seen in humans in consequence of the dramatic shrinkage of the lower mandible produced by neotony [73] and the changes closely associated with the move towards a soft diet [74]. Finally teeth are sensitive to influences experienced during adult life, particularly those nutritional, metabolic, traumatic events and diseases that lead to changes in colouration (e.g. [54], [55]).

Age also has an important influence on the appearance of teeth, with these tending to become yellower and darker as people get older [57], [58], [59], [60]. This may partly explain why females are more concerned about the appearance of their teeth than men [75]. Age-related changes in colour mean that women’s teeth are also serving to signal residual reproductive value (e.g. [76]). Hence women smile more than men [77], [78], [79] and are the only sex to commonly enhance the prominence of their tooth displays by the wearing of lipstick [80].

The lack of effect of whitening teeth on increasing attractiveness beyond that seen in natural tooth colour is in keeping with other findings (e.g. [81]). However in the present study this could be an artefact of the relatively young age of the participants and the models. The darker and yellower teeth of older women signal lower residual reproductive value than the whiter teeth of younger women. Hence, whilst there may be no advantage in increasing the whiteness of young women’s’ teeth, for whom these are just one of an array of signals indicating their youth and consequent high residual reproductive value, this may not be the case in older women.

Similarly, the generally lower ratings of widely spaced (microdontic) teeth by both sexes when seen in both sexes and in all colour conditions may also be age related. Microdontia is strongly associated with low birth-weight [82] and low normal term birth-weight is strongly associated with increased rates of coronary heart disease, stroke, hypertension, non-insulin dependent diabetes and an early demise [83], [84], [85], [86], [87], [88], [89], [90]. Therefore it may be predicted that microdontia will be viewed even more negatively with increasing age of the model, as the poor health outcomes signalled by this tooth array move temporally closer.

In summary, present findings suggest that digital manipulations of tooth colouration and spacing exert an influence over ratings of attractiveness of both male and female faces. The effects were however most strongly seen in female faces. Therefore it is tentatively concluded that the teeth of both sexes do indeed act as human ornament displays but that the female display is more complex because it additionally signals residual reproductive value, although more studies are required to fully confirm this.

## References

[1] Bonato M, Evans MR, Hasselquist D, Cherry MI (2009) Male coloration reveals different components of immunocompetence in ostriches, Struthio camelus. Anim Behav 77: 1033–1039.

[2] Göransson G, VonSchantz T, Fröberg I, Helgée A, Wittzell H (1990) Male characteristics, viability and harem size in the pheasant, *Phasianus colchicus* . Anim Behav 40: 89–104.

[3] Kraaijeveld K, Kraaijeveld-Smit FJL, Komdeur J (2007) The evolution of mutual ornamentation. Anim Behav 74: 657–677.

[4] Møller AP, Saino N (1994) Parasites, immunology of hosts, and host sexual selection. J Parasitol 80: 850–858. 7799157

[5] Senar JC, Figuerola J, Pascual J (2002) Brighter yellow blue tits make better parents. Proc. R. Soc. B 269: 257–261. 10.1098/rspb.2001.1882 PMC1690890 11839194

[6] Zahavi A (1975) Mate selection- a selection for a handicap. J Theor Biol 53: 205–214. 1195756 10.1016/0022-5193(75)90111-3

[7] Andersson M (1982) Female choice selects for extreme tail length in a widow bird. Nature 299: 818–820.

[8] Massaro M, Davis LS, Darby JT (2003) Carotenoid-derived ornaments reflect parental quality in male and female yellow-eyed penguins (Megadyptes antipodes). Behav Ecol Sociobiol 55: 169–175.

[9] Bendich A (1993) Biological functions of dietary carotenoids. Ann NY Acad Sci 691: 61–67. 8129319 10.1111/j.1749-6632.1993.tb26157.x

[10] Lozano GA (1994) Carotenoids, parasites and sexual selection. Oikos 70: 309–311.

[11] Rock CL, Jacob RA, Bowen PE (1996) Update on the biological characteristics of the antioxidant micronutrients: Vitamin C, vitamin E, and the carotenoids. J Am Diet Assoc 96: 693–702. 8675913 10.1016/S0002-8223(96)00190-3

[12] Hill GE (1990) Female house finches prefer colourful males: Sexual selection for a condition-dependent trait. Anim Behav 40: 563–572.

[13] Hill GE, Montgomerie R (1994) Plumage colour signals nutritional condition in the House Finch. Proc. R. Soc. B 258: 47–52.

[14] Hill GE (1999) Is there an immunological cost to carotenoid-based ornamental coloration? Am Naturalist 154: 589–595. 10561131 10.1086/303264

[15] Feinberg DR, Jones BC, DeBruine LM, Moore FR, Law Smith MJ, et al (2005) The voice and face of woman: One ornament that signals quality. Evol Hum Behav 26: 398–408.

[16] Singh D (1993) Adaptive significance of female physical attractiveness: Role of waist-to-hip ratio. J Pers Soc Psychol 65: 293–307. 8366421 10.1037//0022-3514.65.2.293

[17] Fink B, Grammer K, Matts PJ (2006) Visible skin color distribution plays a role in the perception of age, attractiveness, and health in female faces. Evol Hum Behav 27: 433–442.

[18] Fink B, Matts PJ, Klingenberg H, Kuntze S, Weege B, et al (2008) Visual attention to variation in female facial skin color distribution. J Cosmet Dermatol 7: 155–161. 18482022 10.1111/j.1473-2165.2008.00382.x

[19] Gangestad SW, Thornhill R (1997) Human sexual selection and developmental stability. In Simpson JA, Kenrick DT, editors. Evolutionary Social Psychology. Mahwah, NJ: Erlbaum. 169–195.

[20] Thornhill R, Moller AP (1997) Developmental stability, disease, and medicine. Biol Rev 72: 497–548. 9375532 10.1017/s0006323197005082

[21] Courtiol A, Raymond M, Godelle B, Ferdy JB (2010) Mate choice and human stature: Homogamy as a unified framework for understanding mate preferences. Evolution 64: 2189–2203. 20199563 10.1111/j.1558-5646.2010.00985.x

[22] Darwin C (1872) The Expression of the Emotions in Man and Animals. London: Murray. 322 p.

[23] Hess U, Beaupré M, Cheung N (2002) Who to whom and why–cultural differences and similarities in the function of smiles. In Abel M, editor. An empirical reflection on the smile. NY: The Edwin Mellen Press. 187–216.

[24] Krumhuber E, Manstead ASR, Cosker D, Marshall D, Rosin PL (2009) Effects of dynamic attributes of smiles in human and synthetic faces. A simulated job interview setting. J Nonverbal Behav 33: 1–15.

[25] Thornton GR (1943) The effect upon judgments of personality traits of varying a single factor in a photograph. J Soc Psychol 18: 127–148.

[26] Godoy R, Reyes-Garcia V, Huanca T, Tanner S, Leonard WR, et al (2005) Do smiles have a face value? Panel evidence from Amazonian Indians. J Econ Psychol 26: 469–490.

[27] Mehu M, Dunbar RIM (2008) Naturalistic observations of smiling and laughter in human group interactions. Behaviour 145: 1747–1780.

[28] Fridlund A (1994) Human facial expression: An evolutionary view. New York, NY: Academic Press. 318 p.

[29] Houser ML, Horan SM, Furler LA (2007) Predicting relational outcomes: An investigation of thin slice judgments in speed dating. Human Communication 10: 69–81.

[30] Koeppel LB, Montagne-Miller Y, O’Hair D, Cody MJ (1993) Friendly? Flirting? Wrong? In Kalbfliesch PJ, editor. Interpersonal communication: Evolving interpersonal relationships. Hillsdale, NJ: Lawrence Erlbaum. 13–32.

[31] Kowalski RM (1993) Inferring sexual interest from behavioural cues: Effects of gender and sexually relevant attitudes. Sex Roles 29: 13–36.

[32] Moore MM (1985) Nonverbal courtship patterns in women: Context and consequences. Ethol Sociobiol 6: 237–247.

[33] Lee H-K, Lee K-D, Merchant AT, Lee S-K, Song K-B, et al (2010) More missing teeth are associated with poorer general health in the rural Korean elderly. Arch Gerontol Geriat 50: 30–33. 10.1016/j.archger.2009.01.005 19230988

[34] Kim J-M, Stewart R, Prince M, Kim S-W, Yang SJ, et al (2007) Dental health, nutritional status and recent-onset dementia in a Korean community population. Int J Geriatr Psych 22: 850–855. 10.1002/gps.1750 17266172

[35] Okamoto N, Morikawa M, Okamoto K, Habu N, Hazakim K, et al (2010) Tooth loss is associated with mild memory impairment in the elderly: The Fujiwara-kyo study. Brain Res 1349: 68–75. 20599812 10.1016/j.brainres.2010.06.054

[36] Stein PS, Desrosiers M, Donegan SJ, Yepes JF, Kryscio RJ (2007) Tooth loss, dementia and neuropathology in the Nun study. J Am Dent Assoc 138: 1214–1322. 10.14219/jada.archive.2007.0046 17908844

[37] Jansson L, Lavstedt S, Frithiof L, Theobald H (2001) Relationship between oral health and mortality in cardiovascular diseases. J Clin Periodontol 28: 762–768. 11442736 10.1034/j.1600-051x.2001.280807.x

[38] Joshipura KJ, Hung HC, Rimm EB, Willett WC, Ascherio A (2003) Periodontal disease tooth loss, and incidence of ischemic stroke. Stroke 34: 47–52. 12511749 10.1161/01.str.0000052974.79428.0c

[39] Yoshida M, Akagawa Y (2011) The relationship between tooth loss and cerebral stroke. Japanese Dental Science Review 47: 157–160.

[40] Hamalainen P, Meurman JH, Keskinen M, Heikkinen E (2003) Relationship between dental health and 10-year mortality in a cohort of community-dwelling elderly people. Eur J Oral Sci 111: 291–296. 12887393 10.1034/j.1600-0722.2003.00055.x

[41] Chatrchaiwiwatana S (2007) Factors affecting tooth loss among rural Khon Kaen adults: Analysis of two data sets. Public Health 121: 106–112. 17005217 10.1016/j.puhe.2006.06.010

[42] Boyce WT, Den Besten PK, Stamperdahl J, Zhan L, Jiang Y, et al (2010) Social inequalities in childhood dental caries: The convergent roles of stress, bacteria and disadvantage. Soc Sci Med 71: 1644–1652. 20870333 10.1016/j.socscimed.2010.07.045 PMC2954891

[43] Selwitz RH, Ismail AI, Pitts NB (2007) Dental caries. Lancet 369: 51–59. 17208642 10.1016/S0140-6736(07)60031-2

[44] Newton JT, Prabhu N, Robinson PG (2003) The impact of dental appearance on the appraisal of personal characteristics. Int J Prosthodont 16: 429–434. 12956500

[45] Bartlett DW, Fares J, Shirodaria S, Chiu K, Ahmad N, et al (2011) The association of tooth wear, diet and dietary habits in adults aged 18–30 years old. J Dent 39: 811–816. 21911033 10.1016/j.jdent.2011.08.014

[46] Van’t Spijker A, Rodrigues JM, Kreulen CM, Bronkhorst EM, Bartlett DW, et al (2009) Prevalence of tooth wear in adults. Int J Prosthodont 22: 35–42. 19260425

[47] Pfeiffer RA (1964) Dominant erbliche akrocephalosyndaktylie. Zeitschrift fur Kinderheilkunde 90: 301–320. 14316612

[48] Naveh Y, Friedman A (1976) Pfeiffer syndrome: Report of a family and review of the literature. J Med Genet 13: 277–280. 957376 10.1136/jmg.13.4.277 PMC1013415

[49] Cerqueira DF, de Souza IP (2008) Orofacial manifestations of Robinow’s Syndrome: A case report in a pediatric patient, Oral Sur Oral Med O. 105: 353–357. 10.1016/j.tripleo.2007.05.032 18061493

[50] Robinow M, Silverman FN, Smith HD (1969) A newly recognized dwarfing syndrome. Am J Dis Child 117: 645–651. 5771504 10.1001/archpedi.1969.02100030647005

[51] Tosun G, Elbay U (2009) Rapp-Hodgkin syndrome: Clinical and dental findings. J Clin Ped Dent 34: 71–75. 10.17796/jcpd.34.1.kr015833p1qg6873 19953814

[52] Ten Bosch JJ, Coops JC (1995) Tooth color and reflectance as related to light scattering and enamel hardness. J Dent Res 74: 374–380. 7876432 10.1177/00220345950740011401

[53] Sulieman M (2005) An overview of tooth discoloration: Extrinsic, intrinsic and internalised stains. Dent update 32: 463–471. 16262034 10.12968/denu.2005.32.8.463

[54] Joiner A (2004) Tooth colour: A review of the literature. J Dent 32: 3–12. 10.1016/j.jdent.2003.10.013 14738829

[55] Watts A, Addy M (2001) Tooth discoloration and staining: A review of the literature. Brit Dent J 190: 309–316. 11325156 10.1038/sj.bdj.4800959

[56] Morley J (1997) The esthetics of anterior tooth ageing. Curr Opin Cosmet D 4: 35–39. 9663048

[57] Goodkind RJ, Schwabacher WB (1987) Use of a fiberoptic colorimeter for an in vivo color measurement of 2830 anterior teeth. J Prosthet Dent 58: 535–542. 3479551 10.1016/0022-3913(87)90380-5

[58] Hassan AK (2000) Effect of age on colour of dentition of Baghdad patients. East Med Health J 6: 511–513. 11556046

[59] Jahangiri L, Reinhardt SB, Mehra RV, Matheson PB (2002) Relationship between tooth shade value and skin color: An observational study. J Prosthet Dent 87: 149–152. 11854669 10.1067/mpr.2002.121109

[60] Odioso LL, Gibb RD, Gerlach RW (2000) Impact of demographic, behavioural, and dental care utilization parameters on tooth color and personal satisfaction. Comp Cont Educ Dent 29: S35–S41. 11908408

[61] Schmidt CJ, Tatum SA (2006) Cosmetic Dentistry. Curr Opin Otolaryngology & Neck Surgery 14: 254–259. 10.1097/01.moo.0000233596.68928.39 16832182

[62] Alkhatib MN, Holt R, Bedi R (2004) Prevalence of self-assessed tooth discolouration in the United Kingdom. J Dent 32: 561–566. 15386863 10.1016/j.jdent.2004.06.002

[63] Qualtrough AJE, Burke FJT (1994) A look at dental esthetics. Quintessence Int 25: 7–14. 8190886

[64] Xiao J, Zhou XD, Zhu WC, Zhang B, Li JY, et al (2007) The prevalence of tooth discolouration and the self-satisfaction with tooth colour in a Chinese urban population. J Oral Rehabil 34: 351–360. 17441876 10.1111/j.1365-2842.2007.01729.x

[65] Vident website. Available: http://vident.com/products/shade-management/vita-classical-previously-the-lumin-vacuum-shade-guide/. Accessed 2012 Dec 20.

[66] Ekman P, Friesen WV, O’Sullivan M (1988) Smiles When Lying. J Pers Soc Psych 54: 414–420. 10.1037//0022-3514.54.3.414 3361418

[67] Preuschoft S, van Hooff JARAM (1997) The social function of “smile” and “laughter”: Variations across primate species and societies. In Segerståle U, Molnár P, editors. Nonverbal communication: Where nature meets culture. Mahwah, NJ: Erlbaum. 171–190.

[68] Baydas B, Oktay H, Metin Dagsuyu I (2005) The effect of heritability on Bolton tooth-size discrepancy. Eur J Orthod 27: 98–102. 15743869 10.1093/ejo/cjh088

[69] Garn SM, Lewis AB, Kerewsky RS (1965) X-linked ınheritance of tooth size. J Dent Res 44: 439–441. 14278205 10.1177/00220345650440022201

[70] Kotsomitis N, Dunne MP, Freer TJ (1996) A genetic aetiology for some common dental anomalies: A pilot twin study. Aust Orthod J 14: 172–178. 9528418

[71] Brook AH (2009) Multilevel complex interactions between genetic, epigenetic and environmental factors in the aetiology of anomalies of dental development. Arch Oral Biol 54: S3–S17. 19913215 10.1016/j.archoralbio.2009.09.005 PMC2981858

[72] Brook AH (1984) A unifying aetiological explanation for anomalies of human tooth number and size. Arch Oral Biol 29: 373–378. 6611147 10.1016/0003-9969(84)90163-8

[73] Penin X, Berge C, Baylac M (2002) Ontogenetic study of the skull in Modern Humans and the Common Chimpanzees: Neotenic Hypothesis reconsidered with a Tridimensional Procrustes Analysis. Am J Phys Anthropol 118: 50–62. 11953945 10.1002/ajpa.10044

[74] Verhaegen M, Munro S, Vaneechoutte M, Bender-Oser N, Bender R (2007) The original econiche of the genus Homo: Open plain or waterside? In Munoz SI, editor. Ecology Research Progress. New York: Nova Science Publishers. 155–186.

[75] Vallittu PK, Vallittu ASJ, Lassila VP (1996) Dental aesthetics- a survey of attitudes in different groups of patients. J Dent 24: 335–338. 8916647 10.1016/0300-5712(95)00079-8

[76] Marlowe F (1998) The nubility hypothesis: The human breast as an honest signal of residual reproductive value. Human Nature 9: 263–271. 26197484 10.1007/s12110-998-1005-2

[77] DeSantis M, Sierra N (2000) Women smiled more often and openly than men when photographed for a pleasant public occasion in 20th century United States society. Psychology: A Journal of Human Behavior 37: 21–31.

[78] Hall JA, Halberstadt AG (1986) Smiling and gazing. In: Hyde JS, Inn MC, editors. The psychology of gender: Advances through meta-analysis. Baltimore, MD: Johns Hopkins University Press. 136–185.

[79] Morse C (1982) College yearbook pictures: More females smile than males. J Psychol 110: 3–6.

[80] Clarke LH, Bundon A (2009) From ‘the thing to do’ to ‘defying the ravages of age’: Older women reflect on the use of lipstick. J Women Ageing 21: 198–212. 10.1080/08952840903054757 20183145

[81] Höfel L, Lange M, Jacobsen T (2007) Beauty and the teeth: Perception of tooth color and its influence on the overall judgment of facial attractiveness. Int J Periodont Rest 27: 349–57. 17726991

[82] Fearne JM, Brook AH (1993) Small primary tooth-crown size in low birth weight children. Early Hum Dev 33: 81–90. 8055779 10.1016/0378-3782(93)90203-7

[83] Barker DJP (2004) The developmental origins of adult disease J Am Coll Nutrit. 23: S588–S595. 10.1080/07315724.2004.10719428 15640511

[84] Barker DJP, Osmond C, Winter PD, Margetts B, Simmonds SJ (1996) Weight in infancy and death from ischaemic heart disease. Lancet 334: 577–580. 10.1016/s0140-6736(89)90710-1 2570282

[85] Frankel S, Elwood P, Sweetnam P, Yarnell J, Smith GD (1996) Birthweight, body mass index in middle age, and incident coronary heart disease. Lancet 348: 1478–1480. 8942776 10.1016/S0140-6736(96)03482-4

[86] Hales CN, Barker DJP, Clark PMS, Cox LJ, Fall C, et al (1991) Fetal and infant growth and impaired glucose tolerance at age 64. BMJ 303: 1019–1022. 1954451 10.1136/bmj.303.6809.1019 PMC1671766

[87] Leon DA, Lithell HO, Vagero D, Koupilova I, Mohsen R, et al (1998) Reduced fetal growth rate and increased risk of death from ischaemic heart disease: Cohort study of 15 000 Swedish men and women born 1915–29. BMJ 317: 241–245. 9677213 10.1136/bmj.317.7153.241 PMC28614

[88] Osmond C, Barker DJP, Winter PD, Fall CHD, Simmonds SJ (1993) Early growth and death from cardiovascular disease in women. BMJ 307: 1519–1524. 8274920 10.1136/bmj.307.6918.1519 PMC1679586

[89] Rich-Edwards JW, Stampfer MJ, Manson JE, Rosner B, Hankinson SE, et al (1997) Birth weight and risk of cardiovascular disease in a cohort of women followed up since 1976. BMJ 315: 396–400. 9277603 10.1136/bmj.315.7105.396 PMC2127275

[90] Stein CE, Fall CHD, Kumaran K, Osmond C, Cox V, et al (1996) Fetal growth and coronary heart disease in South India. Lancet 348: 1269–1273. 8909379 10.1016/s0140-6736(96)04547-3

